# Fascia Lata Free Flap Reconstruction of Limited Hard Palate Defects

**DOI:** 10.7759/cureus.2356

**Published:** 2018-03-21

**Authors:** Rhorie P Kerr, Andrea Hanick, Michael A Fritz

**Affiliations:** 1 Head and Neck Institute, Cleveland Clinic

**Keywords:** palate, reconstructive surgery, fascia lata, free flap, palate reconstruction, palatal fistula

## Abstract

Objective

The anterior-lateral thigh (ALT) free flap is a flexible reconstructive option with fascia lata, fasciocutaneous, and musculocutaneous options. The objective of this study is to evaluate ALT fascia lata free flap reconstruction of isolated hard palate defects.

Methods

Retrospective chart review of all palate reconstructions with ALT free flap from 2008-2017 by a single surgeon, at a tertiary academic institution. Patients with defects limited to the hard palate were selected for review.

Results

Forty-eight patients were identified, of which 14 patients had limited palatal defects repaired with fascia lata free flaps and were selected for review. The average hospital stay for all patients was 2.8 days (range 1-4 days). Eighty-five percent of patients were started on an oral diet from post-operative day (POD) one. Ten of 14 were extubated at the end of the case, with four being extubated on POD one. One patient suffered donor site morbidity, which required intervention (one seroma requiring drainage). Two patients underwent minor palatal revisions with local tissue rearrangement for recurrent fistula. No patients suffered long-term velopharyngeal inadequacy (VPI) or dysphagia, and all reported normal nasal respiration.

Conclusion

The ALT fascia lata free flap is a versatile reconstructive option for hard palate defects, with minimal morbidity, short hospital stays, and excellent long-term results.

## Introduction

Reconstructive options for standard head and neck defects have advanced steadily, while the palatal and maxillectomy defect has continued to present a varied challenge to the reconstructive surgeon. There remain a variety of distinct approaches for managing the maxillectomy defect, including obturator prosthesis placement, local tissue rearrangement, and a variety of free flap reconstructive options [1-2]. The challenge and general lack of agreement and uniformity with classifying defects involving the palate and maxilla exacerbate this difficulty.  

There exists a significant volume of literature discussing the various surgical reconstructive techniques for palatal defects. Local tissue rearrangement (palatal flaps), various pedicled flaps, and a variety of free tissue transfer methods have all been described. Common free flap options include the radial forearm free flap, anterior-lateral thigh flap (ALT), and fibula and osteocutaneous forearm flaps for larger bony defects. However, there has been little literature discussing the roles of free flap reconstructive techniques in limited palate or maxillectomy defects. Some reconstructive surgeons consider the morbidity, operative time, and hospital stay contraindications for free flap reconstruction for these smaller defects and thus, recommend obturator rehabilitation.

Here, we describe the use of isolated fascia lata (FL) free flap for the reconstruction of limited hard palate and maxillectomy defects with a focus on short hospital stays and minimal access approaches. To our knowledge, this is the first case series describing this unique use and the long-term results.

## Materials and methods

Institutional review board approval was obtained, and a retrospective chart review was completed of all palatal reconstructions performed with the fascia lata and fasciocutaneous antero-lateral thigh free flap from 2008 to 2017, by the senior author at our institution.

Patient charts were reviewed and those with isolated hard palate defects were selected. Charts were reviewed for pertinent demographic data, extent and classification of defect, reconstructive technique, perioperative course, and follow-up results. Long-term functional results, revision surgeries, and any adjuvant therapy was also noted where appropriate. All patients within this case series underwent primary reconstruction with fascia lata free flap. Fascia lata is used for the hard palate as it mucosalizes rapidly, has limited bulk, and limits hair-bearing tissue within the oral cavity.

Indications for free flap reconstruction were the following: 1) previous radiation or ischemic (eg cocaine) or blast (eg gunshot) mechanism for fistula, 2) failed locoregional attempts at repair, or 3) defects too large for regional flap reconstruction.  

**Figure 1:**
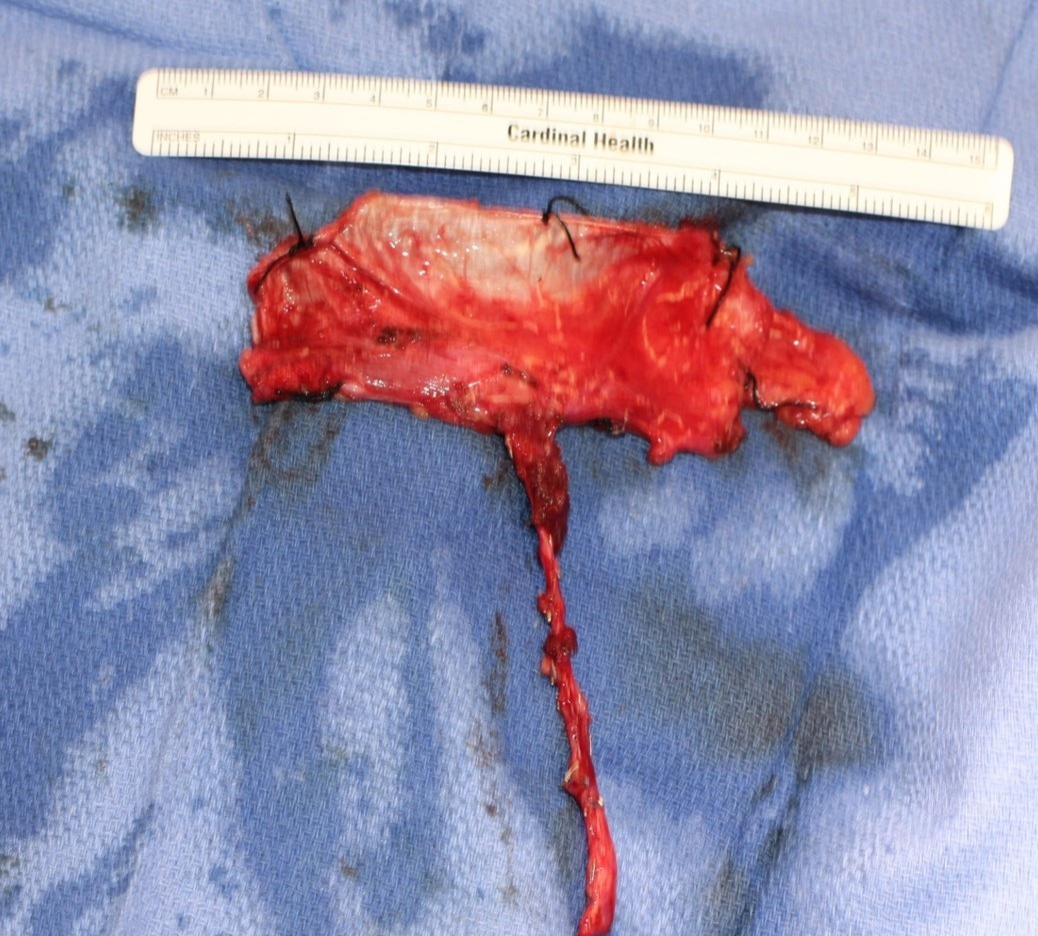
Isolated fascia lata free flap prior to inset

The fascia lata antero-lateral thigh free flap is harvested as a perforator flap containing both fascia and overlying skin, which is left in situ until the pedicle is completely isolated along its length. In our experience, retaining the skin paddle aids in manipulation of the flap during harvest, and prevents damage to the vascular plexus surrounding the fascia lata. Following isolation, the cutaneous and subcutaneous tissues are removed carefully, taking care to preserve the peri-fascial plexus blood supply which is mainly located on the superficial surface of the fascia lata (Figure 1). 

Vascular inset and access are performed in a minimally invasive fashion. As has been previously described, a small incision is made near the facial notch and the facial artery and vein are used for vascular inset. With sufficient pedicle length, the superficial temporal vessels can be used in a similar minimal access technique. The angular vessels, accessed via an incision within the nasolabial sulcus, are also excellent donor vessels that can be accessed with minimal morbidity [3-4].

In all cases, a two-team approach was employed. Recipient site preparation was performed with concurrent free flap harvest to limit operative time.

Patients are typically extubated immediately postoperatively and remain admitted to the hospital until they are ambulating, taking adequate oral intake, and their pain is well-controlled. Typically, patients are started on a liquid diet in the immediate perioperative period and are advanced quickly to full liquid and soft diet within the first 1-2 hospital days.

## Results

Forty-eight patients were identified that underwent anterior-lateral thigh free flap reconstruction of palatal defects. Fourteen patients were identified that underwent fascia lata free flap reconstruction of isolated hard palate defects.

The average patient age was 47 years (range 30 to 69 years of age) and 50% were male (7 of 14). Follow-up time ranged from three months to sixty months at the time of review, with an average follow up time of 24 months. Five patients had a history of radiation therapy to the region, prior to undergoing free flap repair.

The average hospital stay for all patients was 2.8 days (range 1-4 days). One patient left on the day after surgery while five patients left on post-operative day two. Four patients left on post-operative day three, and four patients left on post-operative day four. Twelve patients were started on an oral diet (fully liquid or soft foods) on post-operative day one, while the remaining two were transitioned to an oral diet on post-operative day two. No patients required enteral access or tube feeds during hospitalization.

Ten patients were extubated on post-operative day zero. The remaining four were extubated on post-operative day one.

Two patients required revision surgery. Both of these patients underwent minor, local palatal flap revisions for recurrent fistulas that developed three months and eight months after surgery, respectively. Both of these procedures were performed as same-day procedures without hospitalization. One of these patients requiring a revision procedure had previously had maxillary radiation. Neither of these patients suffered long-term speech or nasal regurgitation side-effects.

At the time of the most recent follow-up, no patients suffered from velopharyngeal insufficiency or nasal regurgitation of oral diet. Of note, no patients within this study underwent any form of speech or swallowing therapy post-operatively (Table 1: Patient Data).

**Table 1:** Tabulated patient data

pt #	Age	Sex	Follow-up Time (months)	Previous Radiation	Revision Surgery Required	Final VPI Status	Final Nasal Regurgitation Status
1	49	f	53	yes	no	None	None
2	69	m	47	yes	no	None	None
3	60	m	23	yes	no	None	None
4	30	m	60	no	no	None	None
5	31	f	1	no	no	None	None
6	37	m	3	no	no	None	None
7	41	m	5	no	no	None	None
8	52	f	20	no	no	None	None
9	53	m	9	yes	yes	None	None
10	30	m	3	no	no	None	None
11	44	f	5	no	no	None	None
12	39	f	12	no	yes	None	None
13	57	f	4	no	no	None	None
14	64	f	1	yes	no	None	None

Morbidity was low in this case series, with no major surgical complications in the early postoperative period. One patient suffered donor site morbidity requiring intervention. This patient developed a seroma 2-3 weeks post-operatively, which was needle aspirated at the second post-operative visit at three weeks.

## Discussion

To date, there have been a large number of studies discussing the various free flap and prosthetic approaches to repairing maxillectomy and palate defects. In addition, there have been multiple reports of the usage of fascia lata for head and neck reconstruction [5-6]. However, there has been little to no literature describing the use of isolated vascularized fascia lata free flap for reconstruction of minor maxillectomy and palatal defects.

**Figure 2:**
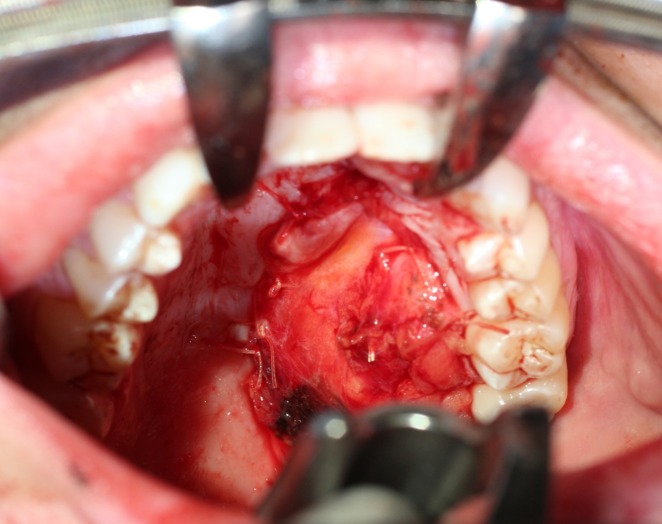
Fascia lata free flap inset

 In our series, we demonstrate the efficacy and low morbidity of the fascia lata free flap for reconstruction of primarily smaller or more limited palatal defects. Our patients all had limited defects, which would often be considered for prosthesis rehabilitation or possibly radial forearm free flap [2, 7]. However, the radial forearm free flap has a number of drawbacks, namely, increased donor site morbidity, hair-bearing tissue in a site not often irradiated postoperatively, and limited flap size. The use of thin vascularized fascia lata allows for repair of palatal defects with limited soft tissue bulk (Figure 2).

**Figure 3:**
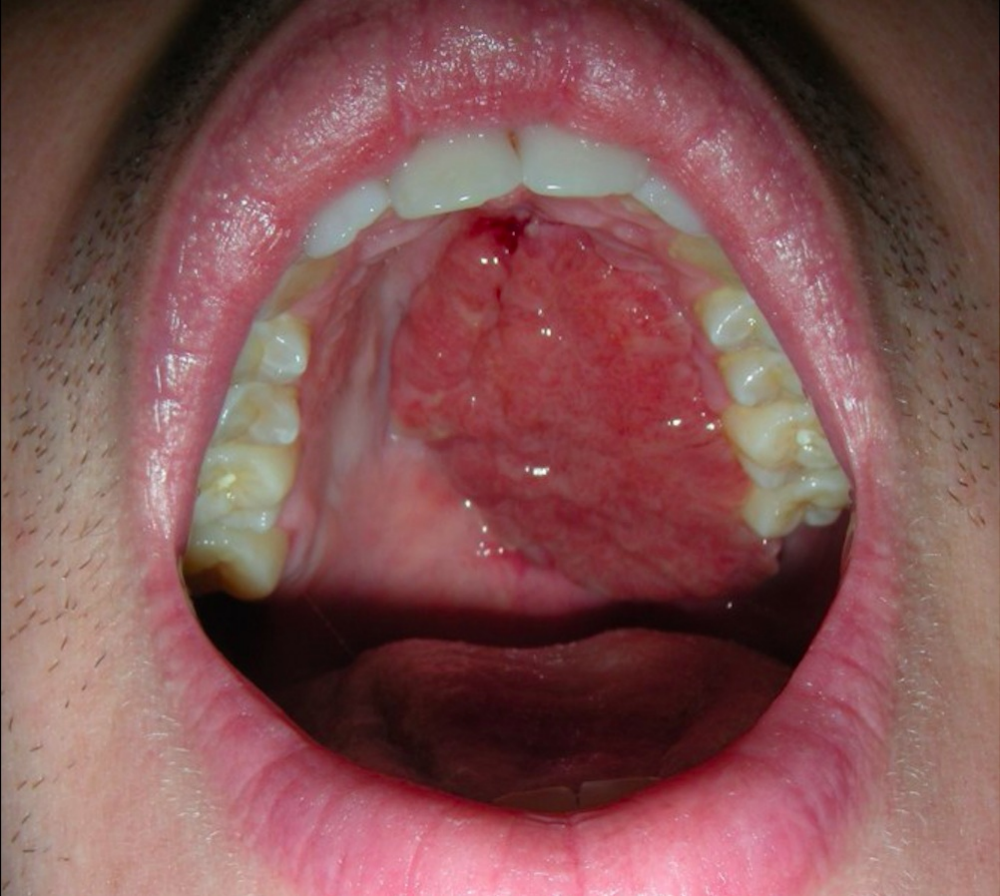
Three weeks postoperative visit demonstrating healthy granulation tissue

**Figure 4:**
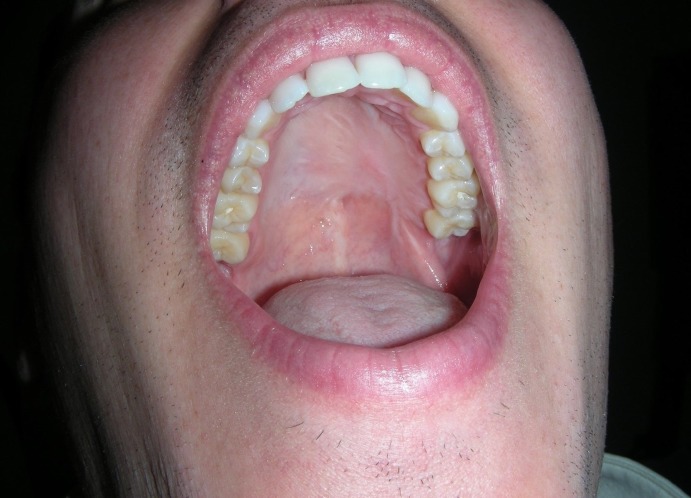
Six months postoperative visit demonstrating well healed free flap

Furthermore, within the oral cavity, the exposed fascia lata granulates and mucosalizes quickly. This obviates the need for cutaneous flaps or skin grafting (Figures 3-4).

Notably, fascia lata tends to contract during healing and so should not be used to primarily repair large soft palate defects. In our experience, a fasciocutaneous ALT free flap is able to adequately repair a complex hard and soft palate defect.

Much of the literature available advocates for prosthetic rehabilitation for smaller defects while reserving free flaps for larger maxillectomy reconstruction. However, prosthetic usage comes with a number of drawbacks; namely, the need for a skilled prosthodontist, frequent cleaning and care, discomfort, and variable results [1-2, 8]. Furthermore, this method still renders the patient dysfunctional when the obturator is not in position, a concern for patients at night, and also as they age and lose both mental faculty and manual dexterity. 

Brandão et al. recently published a systematic review comparing the quality of life outcomes between patients with maxillectomy defects repaired with free tissue transfer and those with defects managed with obturator prosthesis. Based on their review of ten studies, they concluded that the limited data available indicates relatively equal quality of life scores between the two groups [9]. Despite this, there remains a lack of evidence and consensus amongst surgeons regarding the best method of reconstruction. Effective obturator use depends on available residual dentition, a defect of less than half the hard palate and alveolus, a skilled prosthodontist and patient cooperation [2-3].

Obturator function and challenges reported by patients has varied throughout the literature; however, with an appropriately fitting prosthesis patients have generally reported good speech results with little difficulty [1, 10]. Despite this, based on results from Irish et al., up to 20-30% of patients still reported significant difficulties with speech [11]. Within our group of patients, there were no long-term functional deficits in speech or swallowing, highlighting the long-term excellent functional outcomes of this procedure.

Two of our patients required minor revision surgery. Both of these patients initially underwent fascia lata reconstruction for persistent palatal fistula after having failed local reconstructive attempts multiple times. One patient had previously received radiation to the maxilla. Both revision surgeries were for minor recurrent fistulas which were addressed with local tissue rearrangement that did not require hospitalization. Both of these patients have done well with excellent functional outcomes now one and two years out from revision surgery, respectively.

One argument for the use of obturators for reconstruction is the short recovery time. The fascia lata free flap technique requires a relatively short recovery time, as demonstrated by short hospital stays and a quick return to an oral diet. With an average hospital stay of 2.8 days and 85% of our patients starting a significantly oral diet on post-operative day one, this flap technique is unique in its minimal donor site morbidity and rapid return to function.

All of the patients in this series underwent minimal access incisions for microvascular anastomosis. The minimal access incisions for facial vessels, angular vessels, and superficial temporal vessels represent excellent options for both decreasing operative time and obtaining excellent long-term cosmesis. Various studies have demonstrated comparable and favorable flap survival when this approach is employed [7-8].

A two-team approach further limits operative times. In cases where oncologic ablation is required, a two-team approach can greatly benefit operative times, but even in cases without oncologic ablation, a recipient prep team and a free flap harvest team can decrease operative times and limit the traditional length of free flap surgery.

The limitations of this study are the small sample size, lack of objective quality of life and functional assessments, as well as those inherent to retrospective chart reviews. Application of standardized objective measurements for velopharyngeal insufficiency, dysphagia, and quality of life for all future patients undergoing this procedure and larger numbers will increase the applicability of this data. However, the purpose of this study is to present this novel technique and discuss its functional success. Further studies are needed to objectively compare this method to the obturator and other free flap reconstructive techniques. 

## Conclusions

The fascia lata free flap reconstruction of the palate for complex limited palatomaxillary defects represents a novel and successful reconstructive option. With a two-team approach, minimal donor site morbidity, minimal access incisions, and good long-term functional results, this reconstructive method presents a viable alternative to obturation and more traditional reconstructive techniques.
